# Neurosurgical Hyponatremia

**DOI:** 10.3390/jcm3041084

**Published:** 2014-10-14

**Authors:** Mark J. Hannon, Christopher J. Thompson

**Affiliations:** 1 Department of Endocrinology, St. Bartholomew’s Hospital, London, EC1A 7BE, UK; 2 Academic Department of Endocrinology, Beaumont Hospital/RCSI Medical School, Dublin, Ireland; E-Mail: christhompson@beaumont.ie

**Keywords:** hyponatremia, neurosurgery, SIADH, SAH, TBI

## Abstract

Hyponatremia is a frequent electrolyte imbalance in hospital inpatients. Acute onset hyponatremia is particularly common in patients who have undergone any type of brain insult, including traumatic brain injury, subarachnoid hemorrhage and brain tumors, and is a frequent complication of intracranial procedures. Acute hyponatremia is more clinically dangerous than chronic hyponatremia, as it creates an osmotic gradient between the brain and the plasma, which promotes the movement of water from the plasma into brain cells, causing cerebral edema and neurological compromise. Unless acute hyponatremia is corrected promptly and effectively, cerebral edema may manifest through impaired consciousness level, seizures, elevated intracranial pressure, and, potentially, death due to cerebral herniation. The pathophysiology of hyponatremia in neurotrauma is multifactorial, but most cases appear to be due to the syndrome of inappropriate antidiuretic hormone secretion (SIADH). Classical treatment of SIADH with fluid restriction is frequently ineffective, and in some circumstances, such as following subarachnoid hemorrhage, contraindicated. However, the recently developed vasopressin receptor antagonist class of drugs provides a very useful tool in the management of neurosurgical SIADH. In this review, we summarize the existing literature on the clinical features, causes, and management of hyponatremia in the neurosurgical patient.

## 1. Introduction

Hyponatremia is the most frequent electrolyte imbalance encountered in hospital inpatients. It is especially frequent in patients suffering from neurosurgical insult or intervention. Around 15% to 20% of patients admitted for Traumatic Brain Injury (TBI) [1,2,3] and over 50% of patients admitted for Subarachnoid Hemorrhage (SAH) [4,5,6] develop hyponatremia. In patients admitted to neurosurgical units with intracranial tumors and hematomas and in patients undergoing pituitary surgery, hyponatremia occurs in between 10% and 20% of patients [7]. As hyponatremia occurs rapidly, over 2–4 days in most of these conditions, it is more likely to be associated with cerebral edema, and therefore to produce symptoms of cerebral irritation.

Moderate to severe hyponatremia is known to increase inpatient mortality [8,9,10,11,12], and recent data suggest that even mild hyponatremia may confer an adverse prognosis in diverse patient groups, including those with pneumonia [13], those in intensive care [14] and those in the community [15,16]. In addition, as acute hyponatremia is associated with reduced conscious level, the development of this complication may impair the ability of the neurosurgical patient to engage with physiotherapy and rehabilitation. Indeed there are data from a number of centers which highlight the prolongation of hospital stay associated with hyponatremia of multiple etiologies [4,17,18,19,20,21].

As hyponatremia occurs so frequently in neurosurgical patients, management is particularly important. A structured approach to the differential diagnosis of the cause of lowered plasma sodium concentrations forms the basis for prompt and correct management of hyponatremia in this patient group. In this review we will explore the pathophysiology of hyponatremia in neurotrauma with particular reference to the role of vasopressin, and provide a brief summary of our recommended management strategies.

## 2. The Clinical Effects of Hyponatremia

The almost universal finding in all cross-sectional studies of patients with hyponatremia is the finding of increased mortality in patients with low plasma sodium concentrations [8,9,10,11,12]. Gill’s study of hospitalized patients with plasma sodium concentration <125 mmol/L showed an overall mortality of 28%, significantly higher than in eunatremic controls (9%) [8]. However, this study also showed a clear gradation of risk of death according to the severity of hyponatremia, with mortality of 50% in patients with plasma sodium concentrations <115 mmol/L. Clayton’s study of patients with severe hyponatremia (<125 mmol/L) showed that the excess mortality in this group extends beyond the time frame of hospital admission, with a mortality of 20% in hospital and 45% within 6 months of follow up [9]. The high mortality in this study was attributed to the mortality associated with the illnesses which precipitated hyponatremia, such as cardiac failure, liver disease and small cell carcinoma of the lung. However, a role for hyponatremia itself was suggested by a Dutch paper, which reported higher mortality rates in hyponatremic patients who did not receive specific treatment for hyponatremia, compared with those who did (37% *vs.* 13%) [10]. This data would strongly suggest that hyponatremia should not be therapeutically ignored, even if the underlying disease process is serious.

Interestingly, published data from cohorts of patients with milder hyponatremia (<137 mmol/L) would suggest that excess mortality is not confined to patients with plasma sodium <125 mmol/L. Patients with mild hyponatremia in the community [15,16,22], with pneumonia [13] and in intensive care [14] have all been shown to have excess mortality compared with patients with normal plasma sodium concentrations. One of the most comprehensive studies of mortality and hyponatremia is a recently published prospective cohort study of 98,411 patients hospitalized at two Boston teaching hospitals between 2000 and 2003 [17]. The authors documented hyponatremia (<135 mmol/L) in 14.5% of patients on admission, and they were able to demonstrate that hyponatremia increased mortality at 1 year and 5 years. The risk of death was apparent even in those with mild hyponatremia (130–134 mmol/L, hazard ratio 1.38, 95% CI 1.32–1.46).

Although the symptoms associated with hyponatremia are varied and generally related to the severity of hyponatremia, the rate of change in plasma sodium concentration is also fundamentally important to the risk of hyponatremia causing cerebral irritation. Symptoms are far more likely if the fall in plasma sodium is rapid, and tend to occur at higher plasma sodium concentrations [23]. Chronic hyponatremia may present as a relatively asymptomatic condition, even in cases where hyponatremia is severe, due to the presence of cerebral adaptive mechanisms. The initial adaptive mechanism is the loss of intracerebral fluid, with depletion of sodium and potassium [24]. Later, glutamate, myo-inositol, *N*-acetylaspartate, aspartate, creatine, taurine, γ-aminobutyric acid and phosphoethanolamine are lost from the brain, further decreasing intracerebral osmolality and preventing the development of cerebral edema [25]. In contrast, acute hyponatremia rapidly widens the osmotic gradient across the blood-brain barrier, promoting the shift of water into brain cells [26], which generates cerebral edema and rapid neurological deterioration. Unless hyponatremia is corrected promptly and effectively, raised intracranial pressure, cerebral herniation, hypoxia and even death will occur [27], even at plasma sodium levels that may not be considered “dangerous”.

Hyponatremia is particularly common in neurosurgical patients occurring in up to 50% of cases depending on the underlying diagnosis (see Table 1) [7,28]. The incidence is highest following subarachnoid hemorrhage, but hyponatremia is also a common complication of traumatic brain injury, intracranial tumors and hypophysectomy [7]. It is seldom seen in patients treated for spinal lesions [7]. The acute onset of neurosurgical illnesses such as subarachnoid hemorrhage and traumatic brain injury, determines that the development of hyponatremia in the neurosurgical setting is likely to be acute and therefore more likely to be symptomatic. The clinical manifestations of hyponatremia may also be amplified in neurosurgical patients by the co-existence of other factors which cause cerebral irritation, including the original cerebral insult, raised intracranial pressure, or neurosurgical intervention. Furthermore, hyponatremic cerebral irritation is also more likely to occur in patients who have acidosis, hypoxia or hypercapnea, which may accompany acute neurosurgical insults. Therefore some complications, such as hyponatremic seizures, may occur at higher plasma sodium concentrations than might be expected in non-neurological conditions [4]. Data from a number of studies suggest that hyponatremia in the setting of neurosurgical insult is associated with increased duration of hospital stay [4,14,17,28]. More recently it has been shown that inpatient hyponatremia is related to increased mortality in patients with certain neurosurgical illnesses such as intracerebral hemorrhage [29] and ischaemic stroke [30]. Indeed it increasingly appears that any dysnatremia may be a predictor of mortality in neurosurgical patients, with hypernatremia also emerging as a negative prognostic marker [1,6,31].

**Table 1:** Incidence of significant hyponatremia (plasma sodium <130 mmol/L) in patients admitted to the neurosurgical unit in Beaumont Hospital between January 2002–September 2003 (adapted from [7] with permission).

Diagnosis	No. of Patients with Plasma Sodium <130 mmol/L	Total	%
All Patients	187	1698	11
SAH	62	316	19.6
Tumor	56	355	15.8
TBI	44	457	9.6
Pituitary surgery	5	81	6.2
Spinal	4	489	0.81

## 3. The Pathophysiology of Hyponatremia in Neurosurgical Patients

The pathophysiology of hyponatremia in acute brain injury is varied, and sometimes multifactorial (see Table 2). In clinical practice, the differential diagnosis of hyponatremia is further complicated by the practice in many neurosurgical units to use high volumes of intravenous fluids in an attempt to prevent cerebral vasoconstriction. Most studies of the etiology of neurosurgical hyponatremia have been retrospective, and most lack reliable hormonal, hemodynamic and biochemical data with which to inform accurate diagnosis.

**Table 2:** The etiology, diagnosis and basic management of neurosurgical hyponatremia.

Diagnosis	Blood Volume Status	Diagnostic Criteria	Treatment
SIADH	Euvolaemic	See Table 3	Fluid restriction Vaptan therapy
Acute ACTH deficiency	Euvolaemic (may be hypotensive)	0900 h cortisol <300 nmol/L in stressed patient	Steroid replacement therapy
Hypovolaemia	Hypovolaemic	Negative fluid balance	IV fluids
CSWS	Hypovolaemic	Profound diuresis and natriuresis Low BP and CVP	Aggressive IV fluids
Mixed SIADH and CSWS	Variable/fluctuating	Usually SIADH initially, then progressing to CSWS	Depends on stage
Inappropriate IV fluids	Hypervolaemic	Positive fluid balance May have edema/LVF	Diuretics Stop IV fluids

**Table 3:** Diagnostic criteria for the diagnosis of syndrome of inappropriate antidiuretic hormone secretion (SIADH) [32,33].

1. Hyposomolality; plasma osmolality <280 mOsm/kg
2. Inappropriate urinary concentration (Uosm >100 mOsm/kg)
3. Patient is clinically euvolemic
4. Elevated urinary sodium (>40 mmol/L), with normal salt and water intake
5. Exclude hypothyroidism and glucocorticoid deficiency―particularly in patients with neurosurgical conditions

Table 2 emphasizes that the treatment of neurosurgical hyponatremia varies according to the causation of the electrolyte abnormality. The key to correct diagnosis—and therefore appropriate treatment—is an accurate assessment of the blood volume status of the patient. Hypovolemia is most reliably diagnosed when central venous pressure (CVP) monitoring is available, but hypotension, tachycardia, raised plasma urea, and raised plasma renin activity are also useful parameters to consider. Low urine sodium concentration almost always indicates hypovolemia, with consequent secondary hyperaldosteronism and renal sodium conservation. However, the rare condition of cerebral salt wasting is characterized by hypovolaemia with extremely high urine sodium excretion. Hypervolemia leads to an elevated CVP and is also characterized by clinical signs of fluid overload, including peripheral and pulmonary edema as well as a positive fluid balance.

SIADH is characterized by inappropriate water retention at the level of the kidney, leading to a dilutional hyponatremia in a clinically euvolemic patient. Studies in rat models of SIADH have demonstrated that the increase in water reabsorption is secondary to arginine vasopressin (AVP)-mediated expression of renal aquaporin-2 expression [34] with a consequent increase in aquaporin protein excretion in the urine [35]. Prolonged SIADH is associated with “escape” from antidiuresis, with down-regulation of AQP2 mRNA and protein expression. More recently, it has been shown that AVP also regulates water balance and stabilizes fluid osmolality at the cellular level of the brain parenchyma [36]. The pathological event of acute brain injury, ischemia or hypoxia results in an energy deficit for membrane stabilizing ion pumps, and is most pronounced at the site of neuronal synaptic propagation. This leads to osmotic dysbalance between the intra and extracellular compartment which challenges the aquaporin and AVP regulatory systems. Additional systemic hypotension due to cardiovascular regulatory compromise activates the release of vasopressor hormones, among them AVP, leading to hyponatremia due to SIADH.

The diagnostic criteria for SIADH are outlined in Table 3, and it is important to stress that this is largely a clinical diagnosis, supported by biochemical parameters. The patient must be euvolemic with inappropriate urinary concentration. It is also of key importance to distinguish euvolemic hyponatremia due to adrenocorticotrophic hormone (ACTH) deficiency from SIADH in the setting of hyponatremia following neurotrauma. The biochemical presentation of acute glucocorticoid insufficiency is identical to that of SIADH. Patients with ACTH deficiency and hyponatremia have elevated plasma AVP concentrations [37] which may be partially baroregulated due to subtle volume contraction [38]. Furthermore, cortisol itself is required for free water excretion, which also contributes to the development of euvolemic hyponatremia [39]. Glucocorticoid therapy has been shown to suppress AVP secretion and to normalize plasma sodium concentrations in patients with ACTH deficiency [40]. Patients with secondary glucocorticoid deficiency develop hyponatremia with similar frequency and severity as patients with Addison’s disease [41], and acute glucocorticoid insufficiency should be considered as the cause of hyponatremia in any patient with acute neurotrauma. Although guidelines also call for the exclusion of acute thyroid dysfunction to make a diagnosis of SIADH, thyroid function tests in acute illnesses are often misleading, because of the development of sick euthyroid syndrome. Our approach is to perform thyroid function tests only where it is felt that organic thyroid pathology is a significant possibility.

Cerebral salt wasting syndrome (CSWS) is a rare cause of hyponatremia in the setting of neurotrauma. It was first described in 1950 by Peters *et al.* [42] and it has since been described in association with a spectrum of intracranial conditions, including SAH [43], clipping of intracranial aneurysms [44], and TBI [45]. The original researchers hypothesized that the cerebral disease directly attenuated renal sympathetic innervation, causing natriuresis and diuresis, with resultant hyponatremia and blood volume depletion. The possibility of a potential condition separate from SIADH which could cause hyponatremia in neurotrauma was revisited in 1981 after a report of 12 unselected patients, who had developed hyponatremia following subarachnoid hemorrhage, intracranial aneurysm and traumatic brain injury [43]. Hyponatremia developed in association with natriuresis and inappropriate urine concentration in ten patients with good evidence of reduction in plasma volume and total blood volume. Further isolated reports subsequently emerged, detailing the existence of CSWS in neurotrauma [2,46,47], with hyponatremia developing in association with natriuresis and evidence of volume depletion. The evidence to support a syndrome of cerebral salt wasting has not been universally accepted, however, with speculation that the diuresis and natriuresis which characterize CSWS may simply represent escape from antiduresis following SIADH [48]. Many of the studies detailing cases of CSWS rely on retrospective data; the two largest prospective studies to date to examine the etiology of hyponatremia in patients with TBI and SAH, performed by our group, did not find any evidence of CSWS [1,6]. In these two studies, every patient was prospectively evaluated both clinically and biochemically for evidence of volume depletion that would suggest CSWS; no cases were found. CSWS appears to be a rare cause of hyponatremia in the setting of neurotrauma.

## 4. Hyponatremia Following Traumatic Brain Injury 

Following TBI, approximately 15% of patients develop hyponatremia, usually within the first 5 days after cerebral insult [1,3]. Low plasma sodium concentrations in this setting are almost always transient and self-resolving [1,45]. Hyponatremia was previously thought to be primarily due to SIADH [7,49], with acute ACTH deficiency felt to be a relatively rare entity. However, almost all previous studies relied on assessment of cortisol dynamics at a single time point, providing only a “snapshot” of patients’ pituitary function [50,51,52,53,54,55,56]. Plasma cortisol levels are highly dynamic in the days following TBI [51,52], so protocols with only a single time point for testing may underestimate the true incidence of pituitary dysfunction immediately following TBI. A recent prospective study repeatedly and prospectively evaluated for the presence of hyponatremia and acute ACTH deficiency in a large cohort of patients following TBI. The data showed that 15% of patients developed transient hyponatremia. When plasma cortisol concentrations in the TBI patients were compared with those derived from a comparable group of intensive care patients without neurotrauma, 87% of the TBI patients were found to have inappropriately low plasma cortisol concentrations for their degree of illness. Treatment with parenteral hydrocortisone, led to resolution of hyponatremia in all cases [1]. No cases of CSWS were seen despite careful prospective data collection. Thus, acute glucocorticoid insufficiency seems much commoner after TBI than widely appreciated, and it may play a significant role in the development of hyponatremia [57]. Cases of life threatening hyponatremia [58], sometimes associated with hypotension requiring pressor support [54], have been reported which are attributable to acute glucocorticoid deficiency. The presence of hypotension and/or hypoglycaemia are valuable clues which might indicate the presence of acute ACTH insufficiency and, if present in hyponatremic patients, should be further investigated. Long term follow up following TBI has shown that chronic hyponatremia is rare following TBI, and if present another cause (such as treatment with anti seizure medications known to cause hyponatremia) should be sought [1,45].

## 5. Hyponatremia Following Subarachnoid Hemorrhage

Hyponatremia is far more common following SAH than it is after TBI [4,5,6] occurring in approximately 50% of patients. The etiology of hyponatremia following subarachnoid hemorrhage is diverse [2] and potential causes include SIADH, acute glucocorticoid deficiency, and CSWS, as well as the more general causes of hyponatremia outlined in Table 2. However, there is considerable dispute as to which of these diverse aetiologies most commonly cause hyponatremia following SAH. A number of small studies have suggested that CSWS is the most common cause [47,59,60,61], due to the demonstration that there is a rise in plasma concentrations of both atrial natriuretic peptide (ANP) [59,60] and brain natriuretic peptic (BNP) concentrations [62] following SAH. However, these studies were small and underpowered, and not all were able to relate the rise in plasma ANP/BNP concentrations to the subsequent development of hyponatremia. Elevated plasma BNP concentrations may therefore not necessarily mediate the development of hyponatremia. In fact, recent data have suggested that the presence of elevated plasma BNP concentrations cannot be regarded as a reliable predictor of either blood volume status or the development of hyponatremia, within the context of recent subarachnoid hemorrhage [63]. Some previous retrospective studies [4,7] have failed to substantiate cerebral salt wasting as a cause for anything more than a minority of cases of hyponatremia following SAH, and strongly support SIAD as the predominant cause of hyponatremia. In contrast, the similarly-sized retrospective study of Kao *et al*., found that only 35.4% of severe hyponatremia (<130 mmol/L) was considered to be due to SIADH, with a substantial proportion—22.9%—considered to be secondary to CSWS [5]. However, only those patients with plasma sodium <130 mmol/L (a minority of the overall number of cases of hyponatremia) were analyzed in detail. Furthermore, few patients had invasive monitoring of fluid balance parameters such as central venous pressure, and so a diagnosis of CSWS was made based on retrospective review of patients’ fluid balance charts, a method prone to error.

An additional weakness found in the majority of previous retrospective studies is that there was no routine assessment of cortisol dynamics. The authors were therefore unable to comment on how many patients with apparent SIAD had their electrolyte abnormalities as a manifestation of glucocorticoid deficiency. This is a clinically important omission as recent studies by Klose *et al*. [64] and Parenti *et al*. [65] found that between 7.1% and 12% of patients were cortisol deficient immediately following SAH. A recent study involving repeated measurement of plasma cortisol found a far higher rate of ACTH insufficiency [66].

**Figure 1:**
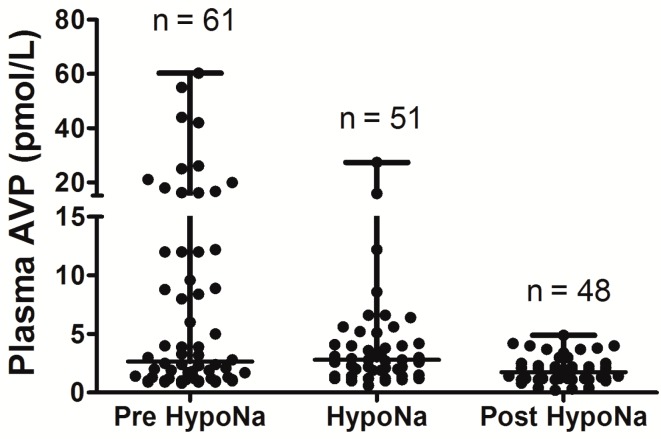
Comparison of arginine vasopressin (AVP) levels before the development of hyponatremia, during the hyponatremic episode, and after resolution of hyponatremia, in patients with SIADH following Subarachnoid Hemorrhage (SAH); AVP levels are significantly higher before and during episode of hyponatremia when compared with after resolution of hyponatremia (*p* = 0.03); adapt from [6] with permission.

**Figure 2:**
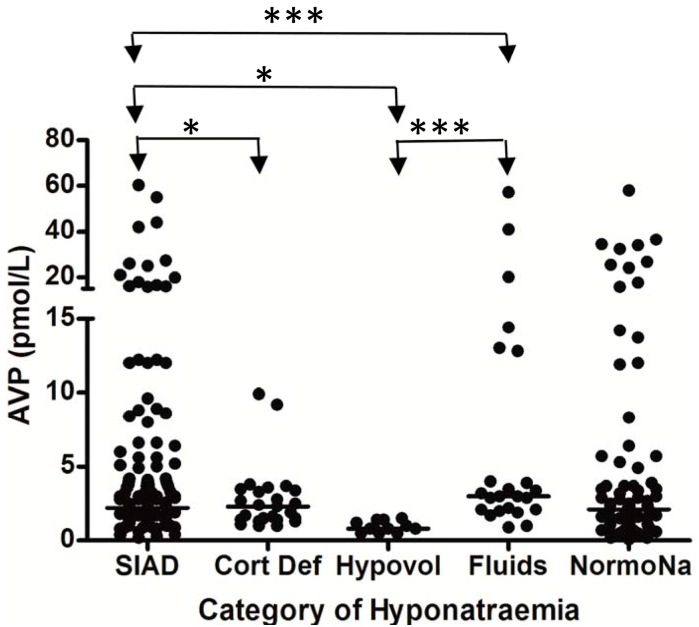
Comparison of AVP levels between different patient groups in patients with hyponatremia following SAH; each point represents an individual AVP measurement; adapt from [6] with permission.

**Figure 3:**
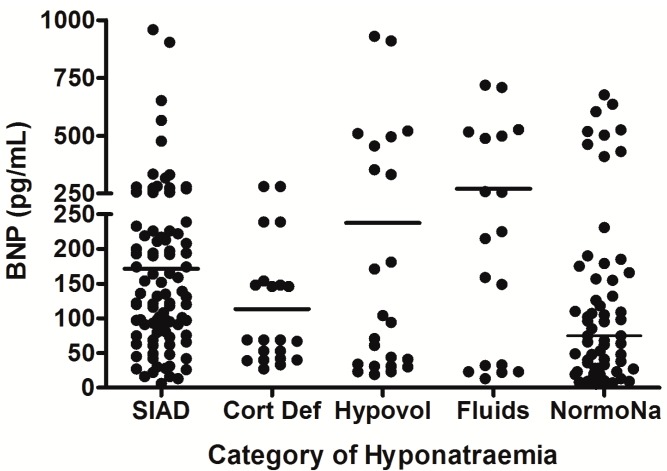
Comparison of brain natriuretic peptic (BNP) levels between different patient groups in patients with hyponatremia following SAH; each point represents an individual BNP measurement; all comparisons between groups were non significant (*p* > 0.05); adapt from [6] with permission.

In the largest prospective study done to date, which involved repeated, sequential assessments of both plasma sodium and cortisol dynamics, hyponatremia occurred in 49% of SAH patients, and the commonest cause of hyponatremia was SIADH (found in 71.4%) [6]. In addition, 8.2% of the hyponatremic patients had acute ACTH deficiency; steroid treatment led to resolution of hyponatremia in these patients. No cases of CSWS were found. This study was further strengthened by the fact that patients also had sequential measurements of AVP and BNP performed, to confirm the cause of hyponatremia. In each patient who developed hyponatremia, AVP was significantly higher before and during the episode of hyponatremia compared with AVP levels measured once the hyponatremia had resolved (see Figure 1), consistent with the majority of cases of hyponatremia being due to SIADH. In contrast, there was no difference in BNP when levels were compared before, during, and after the episode of hyponatremia. Plasma BNP concentrations were also found to be elevated in eunatremic patients, and were not different in the hyponatremic and eunatremic groups. Plasma AVP concentrations were higher in patients with clinical SIADH than in any other patients with hyponatremia, including those with acute glucocorticoid insufficiency, those with hyponatremia due to inappropriate intravenous fluid administration and those with hypovolemic hyponatremia (see Figure 2). In contrast, there was no difference in plasma BNP concentrations between patients with hyponatremia of various etiologies. There was also no difference between plasma BNP concentrations in the eunatremic patients and the hyponatremic patients (see Figure 3). Furthermore, patients with SIADH had loss of the osmotic link between plasma sodium and AVP release whereas eunatremic patients retained this connection—this finding is consistent with the clinical diagnosis of SIADH, as this disorder is characterised by excessive, random secretion of AVP [32,67]. Thus, although BNP was elevated in almost all patients with SAH, AVP was selectively elevated in those with hyponatremia, supporting the authors’ clinical impression that the majority of patients had hyponatremia due to SIADH.

Overall, the data show that SIADH is the most common cause of hyponatremia following SAH, with acute glucocorticoid insufficiency also playing significant role Although CSWS has been reported following SAH [2,4,6], the largest prospective study to date did not find any cases, despite robust methodology and sequential measurement of both AVP and BNP [6], and CSWS seems to be very rare following SAH. None of the above studies demonstrated any significant occurrence of long-term hyponatremia following SAH; as is observed in hyponatremia following TBI, it appears to be a transient phenomenon.

* *p* = 0.01; *** *p* < 0.0001; SIAD = hyponatremia due to SIADH; Cort Def = hyponatremia due to acute glucocorticoid insufficiency; Hypovol = hypovolemic hyponatremia; Fluids = hyponatremia due to inappropriate hypotonic intravenous fluid administration; NormoNa = normal plasma sodium levels following SAH.

SIAD = hyponatremia due to SIADH; Cort Def = hyponatremia due to acute glucocorticoid insufficiency; Hypovol = hypovolemic hyponatremia; Fluids = hyponatremia due to inappropriate hypotonic intravenous fluid administration; NormoNa = normal plasma sodium levels following SAH.

## 6. Hyponatremia Following Pituitary Surgery

Although pituitary tumors themselves rarely cause hyponatremia unless there is untreated glucocorticoid insufficiency, a fall in plasma sodium concentrations has been described to occur following trans-sphenoidal surgery, with an incidence varying from 3% to 25% depending on the series [68,69]. Hyponatremia following pituitary surgery is predominantly due to SIADH (see Table 1 and Table 2) [7]. The presence of preoperative hypopituitarism makes postoperative hyponatremia more likely [69]. Mechanical irritation of the posterior pituitary or the hypophyseal stalk is thought to be the underlying pathophysiological cause of uncontrolled AVP release. In many pituitary units the patients are routinely treated with stress doses of hydrocortisone in the peri-operative period, thus eliminating the possibility of acute glucocorticoid insufficiency as a cause of hyponatremia in this setting. Transient hyponatremia following pituitary surgery may also occur as part of the triple phase response [2]. This is characterized by acute DI, followed by a period of hyponatremia due to release of pre-formed vasopressin from damaged neurones. The initial development of DI usually occurs in the first 48 hours following surgery, and lasts between 24 and 48 hours. Hyponatremia then develops and lasts for several days, as pre-formed vasopressin is released. Eventually, gliosis of the neurones occurs, with the onset of permanent DI. For this reason, DI following pituitary surgery should only be treated with stat doses of ddAVP, as the prescription of a regular dosing schedule could greatly worsen the hyponatremia that would develop as part of the triple phase response.

Hyponatremia occurs relatively rarely in association with other intracerebral pathologies when compared with TBI, SAH and pituitary surgery [2,7]. If hyponatremia occurs, it is often as a result of pituitary stalk irritation or acute glucocorticoid insufficiency caused by surgical intervention rather than the primary disease process.

## 7. Management of Hyponatremia in the Neurosurgical Patient

An important distinction must be made between chronic and acute symptomatic hyponatremia. Many patients with chronic hyponatremia are able to tolerate significant “severe” lowering of plasma sodium concentration (<120 mmol/L), because adaptative mechanisms prevent cerebral edema. On the other hand, rapid falls in plasma sodium concentration—such as those seen in patients with neurotrauma—may produce seizures and diminished levels of consciousness at plasma concentrations (120–125 mmol/L) which present no neurological threat in chronic hyponatremia. Therefore, the term “acute symptomatic” should be used to refer to hyponatremia associated with neurological symptoms, which is almost always associated with acute (<3 days) falls in plasma sodium concentrations. Neurosurgical patients almost always develop hyponatremia quickly, and are more likely develop hyponatremic symptoms at any given sodium level, as a consequence of their underlying cerebral insult, complications of this insult, surgical treatment or comorbid conditions such as hypoxia or hypercapnia. Neurosurgical hyponatremia is therefore generally classified and managed as acute and symptomatic.

### 7.1. Management of Acute Symptomatic Hyponatremia

Neurological symptoms in patients with acute hyponatremia occur as a result of cerebral edema, as water moves from the hypotonic plasma across the blood brain barrier to the relatively hypertonic brain. The clinical manifestations of acute hyponatremia vary from nausea and malaise, to headache, lethargy, obtundation and eventually seizures, coma, and respiratory arrest can occur as the serum sodium concentration progressively falls below 120 meq/L. Noncardiogenic pulmonary edema has also been described [70,71]. Brain herniation remains the greatest risk in acute severe hyponatremia and in patients with intracranial pathology [72].

Acute symptomatic hyponatremia is a medical emergency. Untreated severe hyponatremia, especially when associated with evidence of cerebral irritation, such as seizures, is potentially fatal [23,73] and recovery may occur with permanent brain damage. On the other hand, treatment itself is potentially hazardous, as rapid correction of hyponatremia can expose the patient to the risk of osmotic demyelination. Osmotic demyelination is a dangerous condition characterised by spastic quadriparesis, cranial nerve palsies, pseudobulbar palsy, with behavioral changes, altered cognition and “locked in syndrome”. Osmotic demyelination predominantly affects the pontine area but 10% of cases the cerebellum, thalamus, midbrain and lateral geniculate body are involved [74]. The risk is highest when chronic hyponatremia is rapidly corrected, particularly in patients with chronic alcohol abuse; on the other hand, those with acute severe hyponatremiaare more likely to be symptomatic of their hyponatremia and less likely to be adversely affected by a relatively rapid correction of plasma sodium level. It has traditionally been advised not to correct the plasma sodium by rate more than 0.5 mmol/L/h [75]—in other words, not more than 12 mmol/L over twelve hours. If plasma sodium is corrected more rapidly than this, there is a rapid restoration of intracellular sodium and potassium, but organic solutes may take 5–7 days to normalize. This leads to a hypertonic extracellular compartment which causes extracellular movement of water to the myelin sheath outside the nerve cell. This leads to intramyelinic edema, osmotic endothelial injury and local release of myelinotoxic factors, precipitating oligodendrocyte failure and death [76]. These changes take 2–3 days to develop.

Recent expert panel recommendations have suggested some changes to this traditional management guideline [77]. The first change is that the elevation in plasma sodium does not need to be evenly distributed across the first 24 hours. In fact, as symptomatic hyponatremia is a medical emergency with significant associated morbidity and mortality, there is a need for rapid initial correction to reduce cerebral edema, especially given the fact that those with acute hyponatremia are less prone to osmotic demyelination with treatment than those with chronic hyponatremia. Published experience with hypertonic saline to treat symptomatic acute hyponatremia, has shown that a 5 mmol/L increase in serum sodium can reduce intracranial pressure and resolve the neurological symptoms of herniation by nearly 50% within an hour [78]. Therefore the suggested approach to hyponatremia with neurological symptoms is to elevate the plasma sodium initially by 3–5 mmol/L over 2–4 hours; this will reverse cerebral edema, reduce intracranial pressure and prevent seizures. For severe symptoms, a 100-mL bolus of 3% saline infusion should be given over 10 min, repeated three times if no clinical improvement. This has been a favorable regimen in a small cohort of runners with acute symptomatic exercise-induced hyponatremia [79]. The remainder of the target rise in plasma sodium can occur over 24 hours. For mild to moderate symptoms with a low risk of cerebral herniation, 3% saline infusion is again recommended but at a slower rate of 0.5–2 mL/kg/h.

The second recommendation is a change in the target rise in plasma sodium concentration. There have been reported cases of osmotic demyelination with rises in plasma sodium between 8–12 mmol/L. Although these cases are rare, it is now recommended that the TARGET rise in plasma sodium concentration is <8 mmol/L over 24 hours. In recognition of the difficulty in avoiding overshooting this tight target however, 12 mmol/L was retained as the MAXIMUM rise in plasma sodium which should be accepted. The target rise in plasma sodium concentration when treating patients in at risk groups, such as alcoholics, is recommended to be <6 mmol/L, with a maximum rise of <8 mmol/L [77].

Overcorrection of hyponatremia can be reversed by the administration of intravenous dextrose, parenteral desmopressin, or both. Although there is little evidence base to determine which of these is preferable, re-induction of hyponatremia in animals with rapid overcorrection of hyponatremia has been shown to reduce mortality [80].

The mainstay of diagnosis of osmotic demyelination is clinical suspicion and examination, aided by T1 weighted MRI which may have the classic appearances of a hypointense pons on sagittal imaging but a hyperintense pons on coronal imaging. Prognosis is variable but usually poor, with persistent neurological deficit [70].

### 7.2. Management of Hyponatremia Due to Glucocorticoid Insufficiency

In patients without acute severe hyponatremia, the key to management is an accurate diagnosis of the underlying cause, as outlined in Table 2. However, this may not be easy in clinical practice. The diagnosis of ACTH deficiency is often particularly difficult to establish in the acutely unwell patient. A recent review advocated a random plasma cortisol cut-off of approximately 15 mcg/dL (414 nmol/L) for the diagnosis of ACTH deficiency in intensive care patients (with normal serum binding proteins) [81]. The Critical Care Medicine Taskforce has recommended a very conservative random total plasma cortisol cut-off of <10 mcg/dL (276 nmol/L), or a delta total plasma cortisol of <9 mcg/dL (248 nmol/L) after adrenocorticotrophic hormone (250 mcg) administration, for the formation of this diagnosis [82]. However, this recommendation is intended to cover all intensive care patients and there is a lack of normative data in neurosurgical patients upon which to base a higher cut-off. Furthermore, the administration of adrenocorticotrophic hormone to patients with acute ACTH insufficiency may lead to appropriate adrenal cortisol production by the intact adrenal glands, and therefore lead to a falsely reassuring result. We routinely measure a 0900 h plasma cortisol concentration in all hyponatremic patients who have biochemical and blood volume data to suggest SIADH. In the presence of hypotension and/or hypoglycaemia, we would empirically commence intravenous glucocorticoids pending laboratory analyses. In those in whom there is no strong clinical suspicion of ACTH deficiency, glucocorticoid treatment is only commenced if the 0900 h cortisol is <10.8 mcg/dL (300 nmol/L) [1]. It should be noted, however, that the diurnal variation of plasma cortisol is usually lost in critical illness and so cortisol measurement may not necessarily have to take place at 0900 h. Our policy decision to measure cortisol at 0900 h is based on the fact that this is the time point for which we have the best control values. Treatment with parenteral glucocorticoids will lead to rapid resolution of hyponatremia due to ACTH deficiency. All patients diagnosed with ACTH deficiency in the acute phase of their neurosurgical illness are reassessed with dynamic pituitary function testing between three and six months into their recovery period—prospective studies in TBI patients indicate that acute hormone deficiencies tend to recover by this stage [1,83,84], and long term anterior hypopituitarism is uncommon following SAH [64]. Long term glucocorticoid therapy is only continued in patients who fail dynamic pituitary testing.

### 7.3. Management of Other Causes of Hyponatremia

The majority of cases of hyponatremia due to neurosurgical insult are acute and symptomatic and need emergent management. However, in cases of mild hyponatremia without physiological decompensation, other management strategies may be considered.

The first line management for SIADH is generally fluid restriction. However, in the management of neurotrauma, patients are often aggressively hydrated to avoid cerebral vasospasm and therefore it is often impossible to obtain adequate fluid restriction. Demeclocycline, while well established in the management of SIADH, is not licensed for this indication. It has an unpredictable response rate and erratic onset of action; it can also lead to nephrotoxicity and a photosensitive skin rash. Urea has been shown to be effective in the management of SIADH in a variety of clinical settings [85,86]. A recent uncontrolled retrospective review of urea treatment in patients with SIADH following SAH showed that urea therapy produced a sustained, reliable normalization in plasma sodium level at low cost and with few adverse effects [87]. However, despite treatment with urea, plasma sodium did not normalize for a median of 3 days. Given that the median duration of hyponatremia following SAH is 3 days [6] it is unclear whether this normalization represents a genuine urea effect, as control data were lacking. Urea is unlicensed for this indication, is not widely available and is very unpalatable. Apart from some centers in Europe, particularly Belgium, there is limited experience worldwide with the use of urea, particularly in the neurosurgical setting. Sodium tablets, fludrocortisone and loop diuretics have all been used for SIADH in this setting but there is no physiological rationale for their use [88].

The development of specific vasopressin receptor antagonists represent a novel therapeutic option in SIADH. There are three receptors for vasopressin, of which the V-2 receptors mediate the antidiuretic response. The vasopressin receptor antagonists prevent binding of vasopressin and thereby causing an aquaresis (selective water diuresis), without altering sodium and potassium excretion. At present in the US, two agents, oral tolvaptan and intravenous conivaptan, are approved for clinical use. In Europe, just tolvaptan is approved. There is a growing database of randomized prospective trials showing a predictable and consistent benefit of vaptans over placebo [89,90], though a recent review emphasized the need for head to head trials with other established, though less well validated, treatments for hyponatremia [91]. Data on the specific use of this class of medications in patients with neurotrauma is limited [69,92] but, given that treatment would be of a relatively short duration, the appeal of a physiologic AVP antagonist is obvious. A recent small study which used tolvaptan to treat SIADH following TBI found that it rapidly and safely normalized plasma sodium with no serious adverse effects [93]. A larger study used a single dose of conivaptan in 124 patients with SIADH following TBI and again showed safe and effective responses [94]. However, patients also received other treatments such as hypertonic saline and/or fluid restriction at their physicians’ discretion; neither study represents a true clinical trial with a comparison group receiving placebo or a different management protocol. There are no published data on the use of vaptans in the management of hyponatremia following SAH. We have used short term vaptan therapy following SAH to reverse hyponatremia safely, improve cognitive function, and allow earlier completion of neurorehabilitation and discharge (unpublished observations). There is a clear and pressing need for new studies to specifically analyze the performance of the vaptans when compared with older treatments for SIADH in the neurosurgical setting.

Although the vaptan class of medications have the potential to replace water restriction as first-line therapy in SIADH, their use is limited by cost considerations. Reported side effects are uncommon and although the literature documents the potential for over-correction of plasma sodium level, there have been no documented cases of osmotic demyelination in patients in whom the vaptans have been used appropriately, within clinical guidelines. A recent multicentre trial (TEMPO 3:4) investigating the usefulness of tolvaptan in Polycystic Kidney Disease [95] raised concerns that elevated liver enzymes was more common among patients who received tolvaptan when compared with placebo. The United States Food and Drug Administration (FDA) has issued safety warnings that tolvaptan should not be used for longer than 30 days and should not be used in patients with liver disease, based on the data above. However, this warning is not in effect in Europe.

Despite recent advances in the pharmacotherapy of hyponatremia, it is important to note that the majority of cases of hyponatremia seen in the neurosurgical setting are acute and symptomatic and require management with hypertonic saline. The latest guidelines for the management of hyponatremia advise against using the vaptans to treat severe symptomatic hyponatremia as hypertonic saline provides more evidence-based correction [77]. The vaptans are also not recommended for the treatment of severe but asymptomatic hyponatremia due to insufficient data on the use of vaptans in hyponatremia of this severity [77].

Treatment with intravenous sodium chloride solution is the specific therapy for cerebral salt wasting [96]. It is often necessary to give large volumes to keep up with urinary losses. As CSWS is invariably self-limiting, aggressive treatment with intravenous fluids is usually only required for a few days [97]. Hypovolemic hyponatremia due to other factors such as concurrent diuretic usage also responds to intravascular volume repletion with intravenous fluids.

## 8. Conclusions

Hyponatremia is associated with increased morbidity and mortality in hospital inpatients, and is common in neurosurgical patients. It is an especially frequent occurrence following TBI, SAH, and pituitary surgery. The most common cause of hyponatremia following neurotrauma is SIADH, with acute glucocorticoid insufficiency accounting for a smaller but significant number of cases. CSWS is very rare following neurotrauma. Although hyponatremia is often mild and self limiting, treatment with fluid restriction is generally unsatisfactory. Although the vaptan class of medications offer a novel new approach to the management of SIAD, the use of hypertonic saline is still the treatment of choice for acute symptomatic hyponatremia.
